# NMDA receptors play an important role in postictal potentiation in immature rats

**DOI:** 10.3389/fnsyn.2026.1742164

**Published:** 2026-06-09

**Authors:** Pavel Mares, James L. Burchfiel, Hana Kubova

**Affiliations:** 1 Institute of Physiology, Academy of Sciences of the Czech Republic (ASCR), Prague, Czechia; 2 University of Rochester, Rochester, NY, United States

**Keywords:** cortical stimulation, epilepsy, glutamate ionotropic receptors, immature rat, postictal period, potentiation

## Abstract

**Objective:**

Immediate postictal potentiation (PIP), a unique phenomenon observed in immature animals, may contribute to the enhanced sensitivity of young animals to seizures and epilepsy. PIP deserves more experimental attention because understanding its mechanisms could provide valuable insights into treating these conditions.

**Methods:**

Using the technique of cortical afterdischarges (ADs), we analyzed the effect of glutamate receptor antagonists on PIP in 12-day-old rats. A conditioning AD was recorded, followed 1 min later by a test AD. Then, an antagonist was administered, and 10 min later, another pair of ADs was elicited.

**Results:**

The most pronounced effect was observed with ifenprodil, a selective antagonist of NMDA receptors that contain the GluN2B subunit, which is the dominant form in immature animals. This antagonist not only suppressed the immediate PIP but also converted the condition to an adult-like postictal depression. Post-drug, the test AD was significantly shorter than the conditioning AD. Less pronounced effects were found with non-selective NMDA antagonists, antagonists that preferntially target NMDA receptors containing the GluN2A subunit (which is the dominant form in adult animals), or metabotropic glutamate receptor antagonists. In contrast, AMPA antagonists had their major effects on delayed PIP. This was particularly true for IEM 1460, a selective antagonist of AMPA receptors that lack a functional GluR2 subunit, which is again a form predominant in immature animals.

**Conclusion:**

PIP appears to be a function of the unique structure of glutamate receptors in the immature nervous system, particularly NMDA receptors.

**Highlights:**

- Postictal potentiation (PIP) rather than depression is observed in immature rats.
- Two types of PIP are identified—immediate (up to 5 min) and delayed.
- Immediate PIP is due to NMDA receptors containing the NR2B subunit.
- Delayed PIP is a more complicated phenomenon. Among systems participating in delayed PIP, AMPA receptors play a role.

## Introduction

The highest prevalence of seizures occurs during the neonatal period (Volpe, 2008; Pressler et al., 2021). This is a unique phase of brain development characterized by a predominance of neuronal excitability (Silverstein and Jensen, 2007). While this dominance of excitation over inhibition is believed to be essential for the processes of early brain development, it also renders the brain more susceptible to seizures and increases the chances of recurrent seizures (status epilepticus) (Holmes and Ben-Ari, 2001), as demonstrated with amygdala kindling in immature rats (Moshé and Albala, 1983; Moshé and Albala, 1983).

Furthermore, neonatal seizures (e.g., hypoxic–ischemic encephalopathy as a frequent cause of neonatal seizures) are extremely difficult to treat (Silverstein and Jensen, 2007). Therapies that are effective in older children and adults are largely ineffective in neonates. This is likely due to the distinctive state of hyperexcitability at this stage of development. Therefore, a better understanding of the neuronal mechanisms underlying the enhanced neuronal excitability of the immature brain will not only further our knowledge of early development but also assist in designing more effective treatments for neonatal seizures.

**Figure 1:**
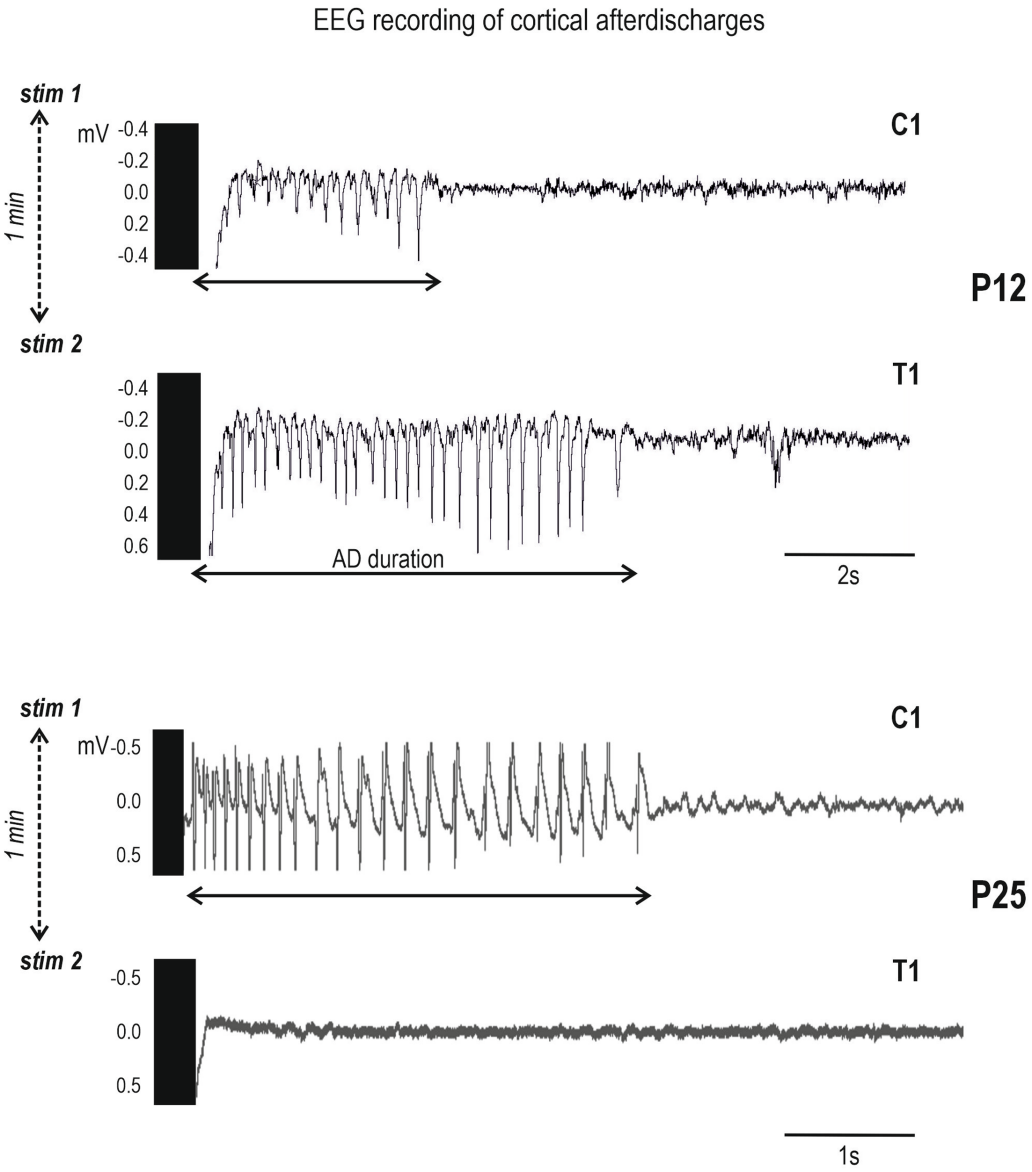
EEG recordings of cortical afterdischarges (ADs) elicited by 15 s of 8-Hz stimulation. Upper two recordings—12-day-old rat, conditioning (C1) and testing (T1) ADs. Interval between the end of conditioning and the testing ADs is 1 min. Lower two recordings for comparison—postictal depression in 25-day-old rat. Time mark in the upper part of figure = 2 s, in the lower part = 1 s.

The cerebral cortical development of a 12-day-old rat is considered comparable to that of a neonatal human (Romijn et al., 1991). In previous studies (Mareš et al., 2002; Mareš and Kubová, 2015), we developed an experimental technique to investigate the hyperexcitability in immature rats. This technique involves applying pairs of identical stimuli to the sensorimotor cortex. The first stimulus (conditioning stimulus) elicits an electrical seizure (afterdischarge [AD]), which is recorded in the contralateral cortex. The second is a test stimulus that assesses neuronal excitability following the initial electrical seizure. The test stimulus elicits a longer AD, indicating enhanced excitability, which we call postictal potentiation (PIP). PIP is unique to the immature stage of development. In older rats, the test stimulus elicits either no AD or a shorter AD. (See Figure 1 for examples.) Therefore, we believe that the experimental phenomenon of PIP serves as an effective model for studying the neuronal mechanisms that contributed to enhanced excitability and seizure susceptibility in the neonatal period.

We investigated the roles of NMDA and AMPA receptors in mediating the hyperexcitability in immature animals. We selected these receptors because of their unique roles in the immature brain. They are overexpressed in immature animals (Insel et al., 1990; Johnston, 1995), and their anatomical composition differs significantly between the immature and more mature brains. NMDA receptors in the immature forebrain have the main NR2 component NR2B, which is replaced during the third week by NR2A. Similarly, there is a time-dependent increase in the GluA2 subunit of AMPA receptors (Stocca and Vicini, 1988; Erdö and Wolff, 1989; Insel et al., 1990; Sheng et al., 1994; Wenzel et al., 1997; Hsieh et al., 2002; Erdö and Wolff, 1989).

Various NMDA receptor antagonists were used. First, we tested non-competitive antagonists of NMDA receptors: dizocilpine (MK-801, Wong et al., 1986) and memantine (a low-affinity antagonist) (Parsons et al., 1993). Then, to determine if a particular subunit is responsible for potentiation, we used specific antagonists: ifenprodil, a selective antagonist active against NMDA receptors containing the GluN2B subunit (Williams, 1993), and PEAQX, an antagonist preferring NMDA receptors with the GluN2A subunit (Auberson et al., 2002). Finally, to indirectly affect the NMDA system, we used MTEP, an antagonist of metabotropic glutamate receptor 5 (Lea and Faden, 2006; Lea and Faden, 2006).

For the AMPA receptor, we tested two antagonists: NBQX, a specific GluA antagonist (Sheardown, 1993), and IEM1460, an antagonist that selectively targets AMPA receptors lacking a functional GluA2 subunit (Magazanik et al., 1997).

We found that PIP in the immature nervous system is due to the unique composition of glutamate receptors present during early development. NMDA receptors appear to play a fundamental role in the hyperexcitability that contributes to seizure susceptibility in neonates. A further, unexpected result raises the possibility of a separate but related role of AMPA receptors in mediating brain changes that increase susceptibility to status epilepticus (for review, Joshi and Kapur, 2024) and enhance long-term susceptibility to epilepsy occurring later in life.

## Methods

### Animals

Twelve-day-old male Wistar strain rats were used for the study. All procedures involving animals and their care were conducted in accordance with the ARRIVE guidelines and national (Act No 246/1992 Coll.) and international laws and policies (EEC Council Directive 2010/63/EU; Guide for the Care and Use of Laboratory Animals, U.S. National Research Council, 1996). The experimental protocol was approved by the Animal Care and Use Committee of the Institute of Physiology and the Central Committee of the Czech Academy of Sciences (No.73-216). All efforts were made to minimize the distress experienced by baby rats (primarily by maintaining their body temperature).

### Stimulation and recording

The procedures followed were consistent with those outlined in previous publications (Mareš and Kubová, 2015; Mareš et al., 2020). Under ether anesthesia, flat silver electrodes were implanted epidurally over the cortex. This anesthesia was used because of its short duration and safety, ensuring maximal survival of the immature rats. A pair of stimulating electrodes was placed over the right sensorimotor cortex (AP −1 and +1, L 2.5 mm), and a recording electrode was placed over the left sensorimotor area (AP 0, L2.5 mm). Reference and grounding electrodes were placed in the occipital bone over the cerebellum.

The electrode assembly was securely attached to the skull with fast-curing dental acrylic. The surgery lasted 10–12 min. Afterward, the animals were allowed to recover for at least 1 h. Righting, placing, and suckling reflexes were tested, and only those animals demonstrating all three reflexes were used for further study. Throughout the experiment, the baby rats were kept on a heating pad maintained at 34 °C. Each animal was used only once. At the end of the experiment, the animals were deeply anesthetized with 3% isoflurane and then decapitated.

Stimulation consisted of 15-s trains of 1 ms biphasic rectangular pulses delivered at 8 Hz from a constant current source. To ensure that the first conditioning stimulation elicited an AD, a suprathreshold stimulation intensity of 6 mA was used. Behaviorally, the animals exhibited clonic movements of the head and forelimbs during the ADs.

We investigated whether various antagonists of NMDA and AMPA receptors could eliminate PIP. The protocol involved two pairs of identical cortical stimuli. Each pair consisted of a conditioning stimulus, followed by a test stimulus1 min later. The first pair served as a control and showed typical PIP. Specifically, the AD elicited by the test stimulus was longer than that triggered by the conditioning stimulus. At the end of the first test AD, an antagonist drug was administered. Then, 10 min later, the second pair of stimuli was applied. If PIP was absent in the second pair, i.e., the test AD was either not present or shorter than the conditioning AD, then this was taken as evidence that the receptor targeted by the antagonist was involved in mediating PIP and, by extension, was an important factor in the hyperexcitability of the immature brain.

### Drugs

The following drugs and doses were used: NMDA receptor antagonists dizocilpine (MK-801, 0.1 and 0.5 mg/kg), memantine (10 and 20 mg/kg), ifenprodil (20 and 40 mg/kg), PEAQX (20 and 40 mg/kg); AMPA receptor antagonists NBQX (7.5, 15, and 30 mg/kg) and IEM1460 (10 and 20 mg/kg); an antagonist of the glutamate metabotropic receptor mGluR5 MTEP (10 and 20 mg/kg). Doses were based on our previous studies of pentylenetetrazol convulsions in immature rats.

Dizocilpine and NBQX were purchased from the Research Institute of Pharmacy and Biochemistry (Prague). Ifenprodil, PEAQX, and IEM1460 were purchased from Tocris (Abingdon, UK). Memantine was purchased from Sigma Aldrich (Prague). MTEP was purchased from Abcam (Cambridge, UK).

The drugs were dissolved in distilled water with the exception of NBQX, which was dissolved in dimethyl sulfoxide (DMSO). All drugs except PEAQX were injected intraperitoneally. PEAQX was given subcutaneously. Each injection takes at most 20 s.

Two control groups were formed—one with physiological saline and the other with DMSO. In addition, to control for potential effects of DMSO, we included a group of animals receiving NBQX suspended in Tween 80 at doses of 7.5 and 15 mg/kg.

### Experimental design

The experimental design is outlined in Supplementary Figure 1 Two pairs of ADs were elicited, separated by 10 min. Each pair consisted of an initial conditioning AD (C1 or C2), followed 1 min later by a test AD (T1 or T2). For each pair, the duration of the test AD was compared to that of the conditioning AD. A longer test AD indicates postictal potentiation (PIP), or a greater state of neuronal excitability elicited by the conditioning AD. A shorter test AD indicates postictal depression, or a diminished state of neuronal excitability elicited by the conditioning AD. Examples of these two different outcomes are illustrated in Figure 1.

Drugs were administered between the two AD pairs, immediately following the end of the T1 AD. The effect of each drug on the state of neuronal excitability elicited by the conditioning AD was determined by comparing the results from the first AD pair (T1 vs. C1) with those from the second AD pair (T2 vs. C2). In all instances, the first pair showed increased neuronal excitability or PIP (i.e., T1 > C1). If the second pair remained unchanged, then the drug had no effect on neuronal excitability. On the other hand, if T2 was less than C2, then the drug abolished PIP, strongly suggesting that the receptor upon which the drug acts is important for mediating the increased neuronal excitability characteristic of immature animals.

### Statistical analysis

Statistical analysis was performed using GraphPad Prism 8 software (GraphPad Software, United States). The program starts by testing the distribution of data. Then, based on these results, it recommends either parametric (ANOVA, t-test) or nonparametric (ANOVA on Ranks, Mann–Whitney test) tests and offers various types of presentation. Sample size was determined in advance according to previous experience with the given models and followed the principles of the three R’s (Replacement, Reduction, and Refinement; https://www.nc3rs.org.uk/the-3rs). Outcome measures and statistical tests were prospectively selected. At the beginning of the study, a simple randomization was used to assign each animal to a particular treatment group. Data acquisition and analysis were done blinded to the treatment. Outliners were identified with the ROUT test (Q = 1%). Using the D’Agostino-Pearson normality test, all data sets were first analyzed to determine whether the values were derived from a Gaussian distribution. Differences in the duration of epileptic afterdischarges (T1 vs. C1, C2 vs. T1, C2 vs. C1, and T2 vs. C2) were analyzed using ordinary RM one-way ANOVA corrected for multiple comparisons by controlling the FDR using a two-stage linear step-up procedure of Benjamini, Krieger, and Yekutieli. A q-value of < 0.05 was required for discovery. A level of significance was set at 5%.

## Results

### Controls

**Figure 2:**
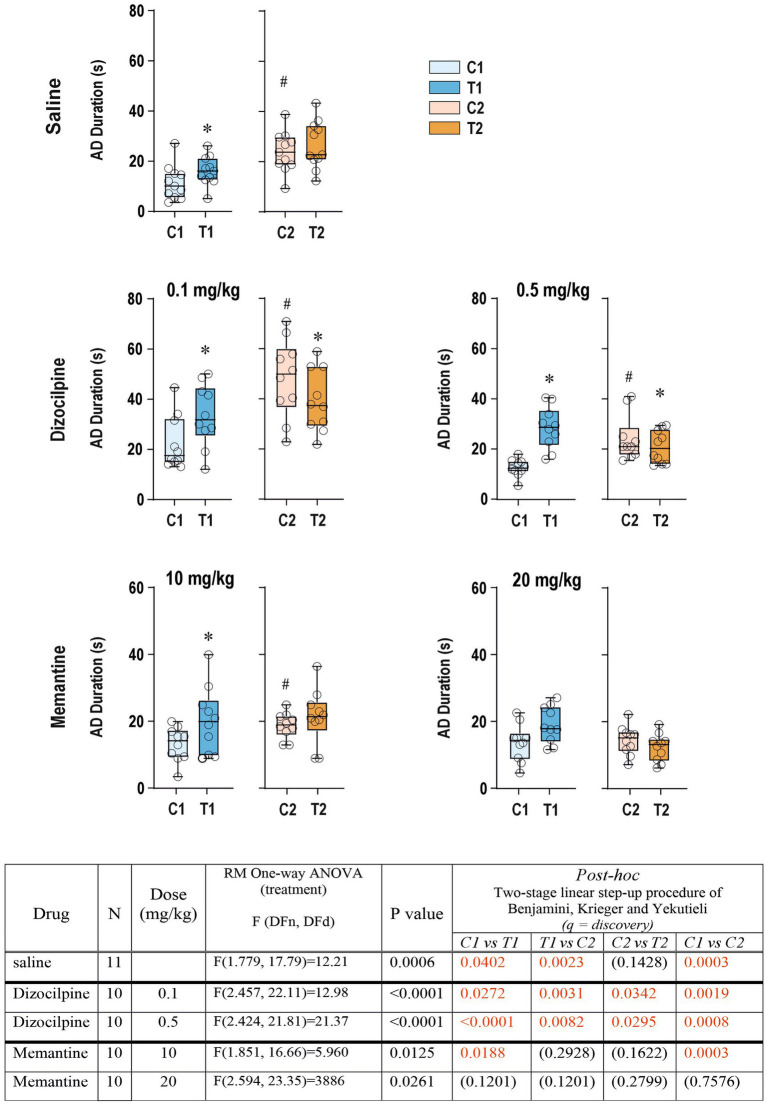
Effects of dizocilpine (MK-801) and memantine on postical potentiation. Data are presented as box plots (the sample median and the 25th and 75th percentile) with whiskers (min and max). Data for presented ADs are in different colours. Individual values are presented as circles. *x*-axis—the four ADs (intervals between C and T are 1 min, between T1 and C2 10 min); *y*-axis—duration of ADs in seconds. Asterisks denote statistical significant difference between corresponding C and T values; double cross between C2 and C1. Lower part of figure—table with statistical data. From left to right: drug; number of animals; dose; result of One Way ANOVA; *p* value; post-hoc comparisons of all four pairs of ADs (value *q*—significant values in red).

The control stimulation trials illustrate the characteristics of postictal potentiation (PIP) in immature animals (Figure 2). In the first pair, the AD elicited by the test stimulus (T1) was significantly longer than the AD elicited by the conditioning stimulus (C1), indicating PIP. The value of T1 is not always significantly longer than C1, but at least a tendency is always present (saline, memantine in a dose of 20 mg/kg, and both doses of dizocilpine). For the second pair, the results are similar but less significant. T2 tended to be longer than C2, but the difference was not always statistically significant. This difference is due to a lingering potentiation effect from the first pair of ADs, even after 10 min. C2 was always significantly longer than C1 and was often longer than T1; therefore, there was little room for further improvement in T2. The important point is that T2 was never shorter than C2, i.e., there was no postictal depression exhibited in the second pair.

### NMDA receptor antagonists

The non-competitive NMDA receptor antagonist, dizocilpine (MK-801), significantly altered the situation (Figure 2). Rather than PIP in the second stimulus pair after administration of the drug, there was postictal depression—T2 was significantly shorter than C2. This strongly suggests that the NMDA receptor mediates PIP. A similar, but smaller, effect was seen with the low-affinity NMDA receptor antagonist, memantine (Figure 2). There was a tendency for T2 to be shorter than C2 at the 20 mg/kg dose, but this was not statistically significant. This lesser effect of memantine in comparison to dizocilpine most likely reflects its lower affinity and dwell time for the NMDA receptor (Parsons et al., 1993).

**Figure 3:**
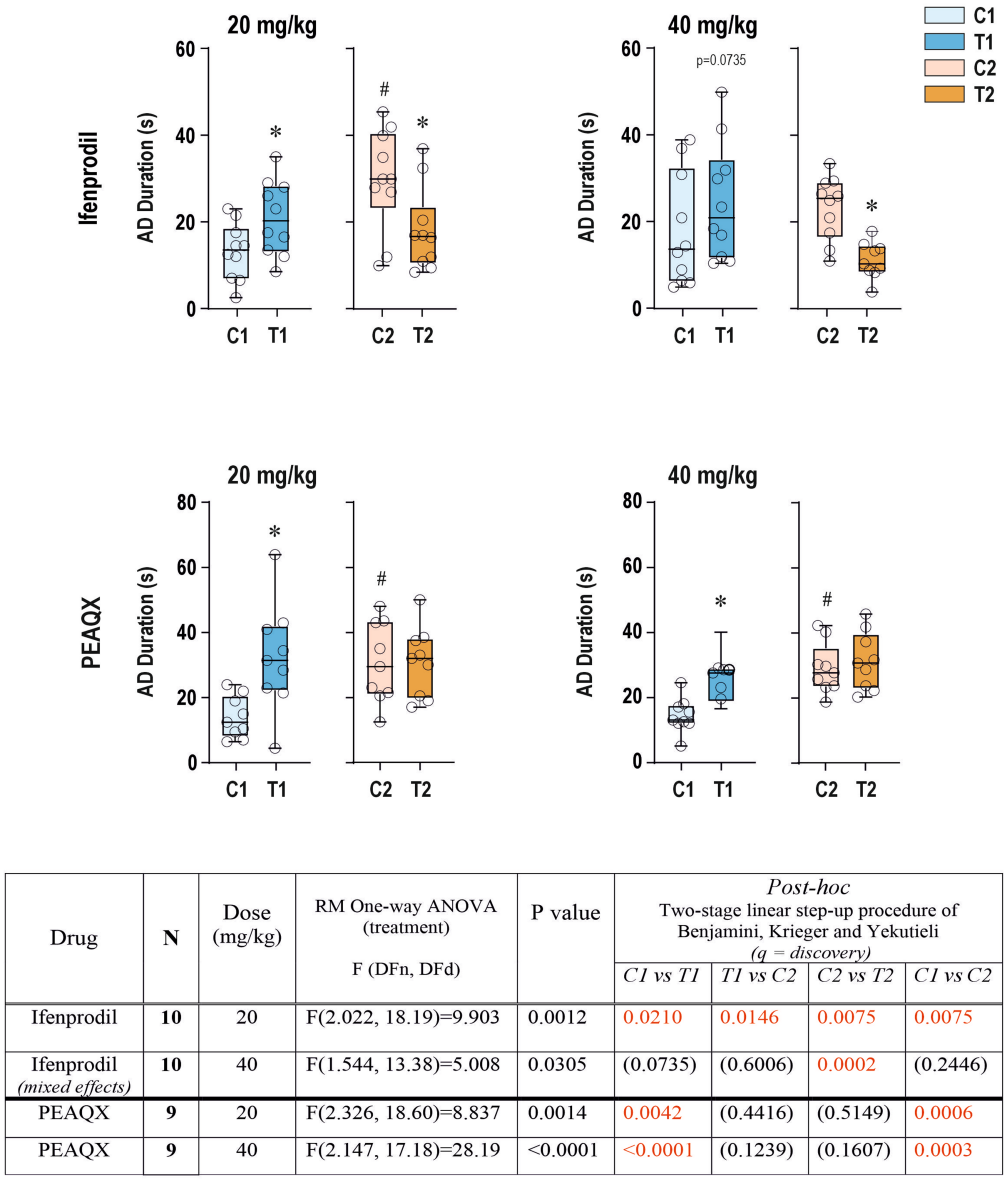
Effects of ifenprodil and PEAQX on postictal potentiation. Details as in Figure 2.

The subunit-specific antagonists further clarified the group of the NMDA receptor involved in PIP. Ifenprodil, the specific antagonist of NMDA receptors containing the GluN2B subunit, also abolished PIP in the second stimulus pair and did so even more dramatically than dizocilpine (Figure 3). After the drug, the PIP seen in the first stimulus pair was converted to postictal depression characteristic of adult animals.

By contrast, PEAQX, a specific antagonist of NMDA receptors containing the GluN2A subunit, was ineffective at abolishing PIP (Figure 3). In the second stimulus pair, T2 equaled C2, as in the control groups. Most significantly, there was no conversion of the immature PIP to adult-type postictal depression.

Finally, MTEP, an antagonist of metabotropic glutamate receptors, also had no effect on PIP (Supplementary Figure 2). Following drug administration, the second stimulus pair showed the same results as the controls, with no difference between T2 and C2.

### AMPA receptor antagonists

**Figure 4:**
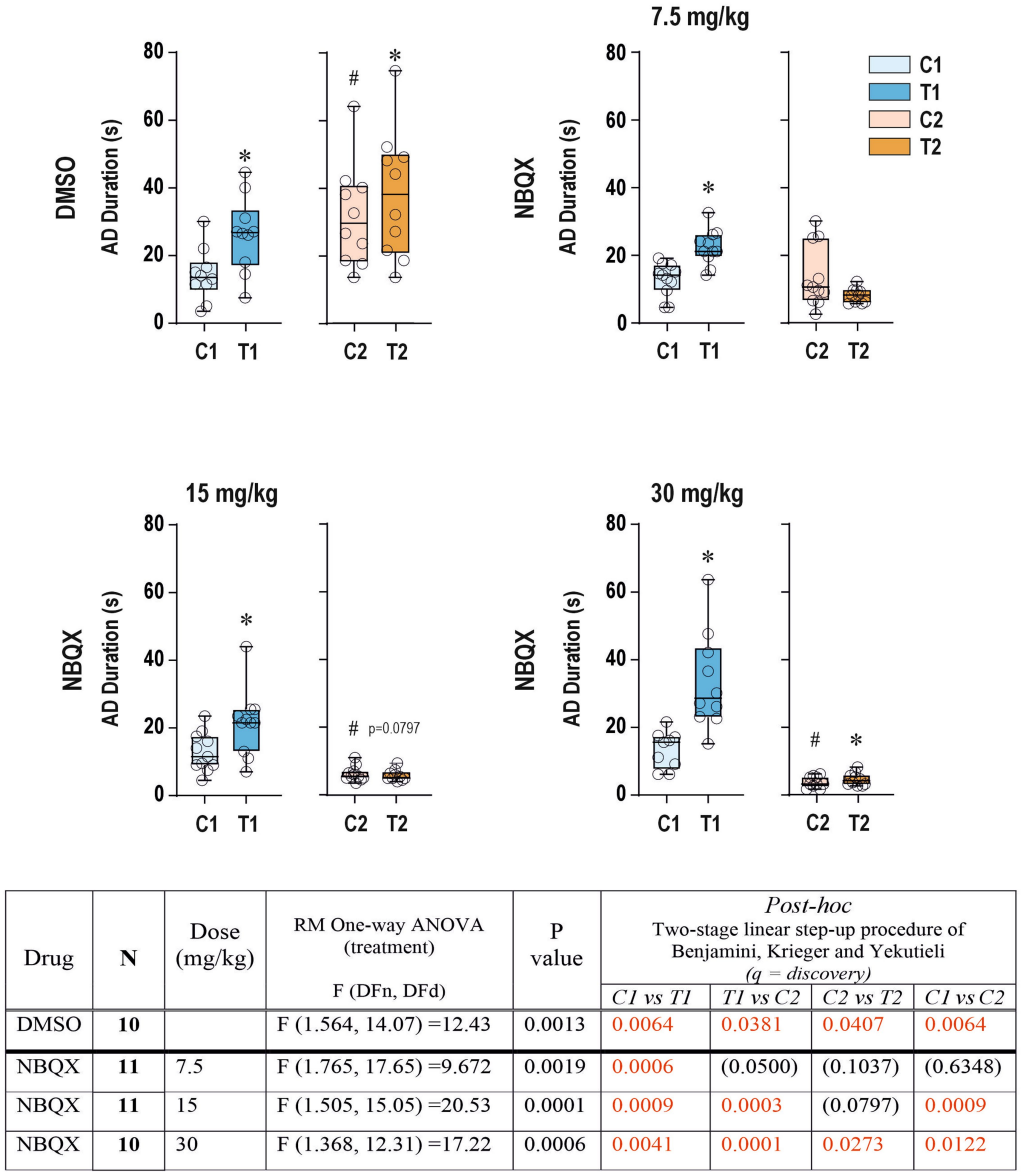
Effects of NBQX dissolved in dimethylsulfoxide on postictal potentiation. Details as in Figure 2.

**Figure 5:**
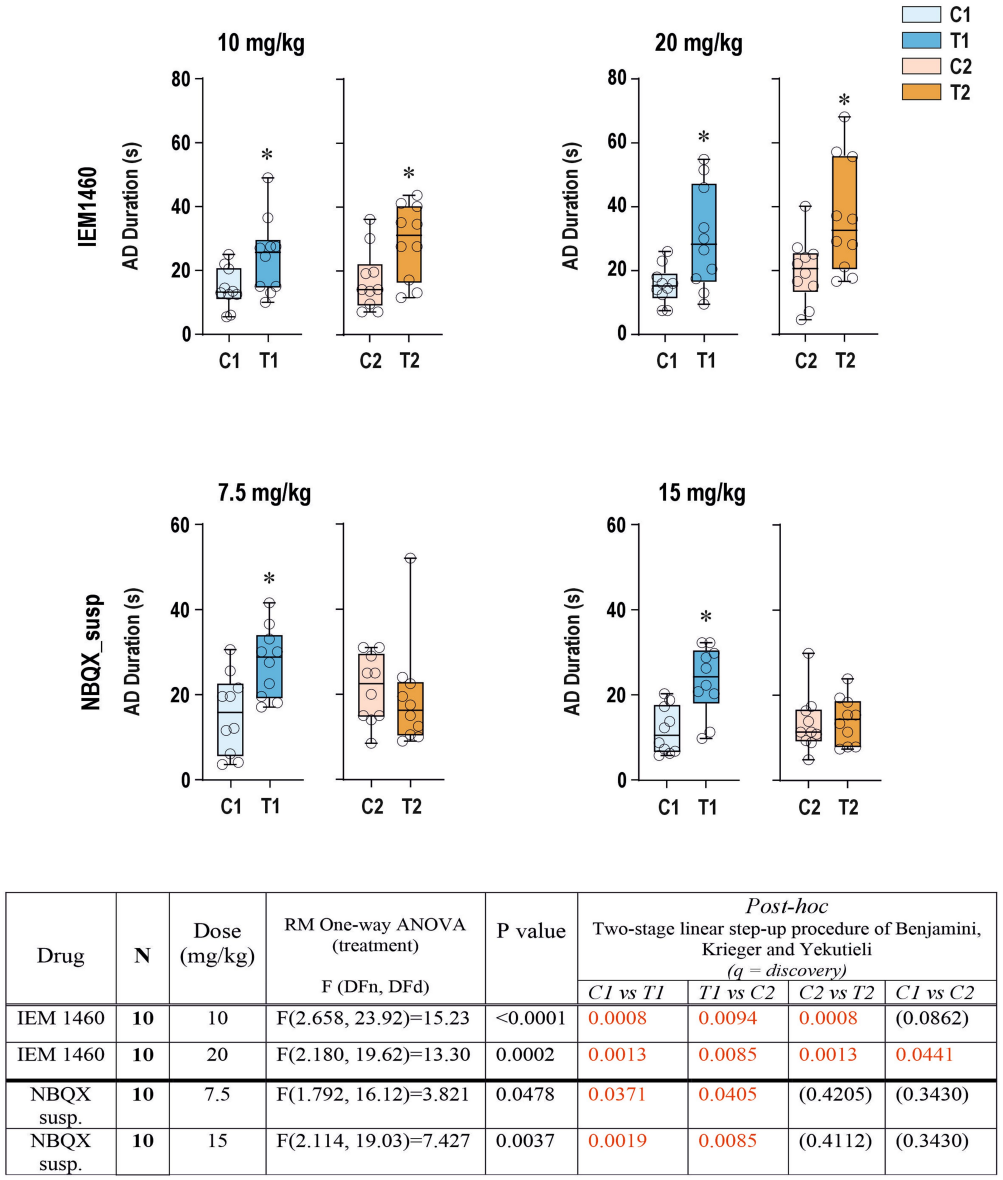
Effects of IEM1460 and NBQX in suspension on postictal potentiation. Details as in Figure 2.

Neither of the AMPA receptor antagonists had a significant effect on the immediate PIP exhibited by 12-day-old rats. Following the administration of either the competitive specific antagonist NBQX (Figures 4, 5—dissolved in DMSO or in suspension), or IEM 1460 (Figure 5), the specific antagonist of AMPA receptors without a functional GluA2 subunit, T2, was not significantly diminished in comparison to C2. Indeed, in IEM 1460, the second stimulus pair showed dramatic PIP similar to that seen in the first stimulus pair.

The AMPA receptor antagonists demonstrated an effect not seen with any of the NMDA receptor antagonists. As we noted earlier in the discussion of the results in control animals, 12-day-old rats tended to show lingering potentiation following the first stimulus pair, where C2 continued to be longer than C1 and, sometimes, was even longer than T1. This prolonged potentiation was abolished by AMPA receptor antagonists. With both drugs, C2 was never longer than C1, and in the case of higher doses of NBQX dissolved in DMSO (Figure 4), C2 was significantly shorter or nearly absent in both DMSO-dissolved and suspension of NBQX.

## Discussion

The neonatal period is a unique stage of development. It is a time of high neuronal excitability (Silverstein and Jensen, 2007). This appears to be an essential factor driving the rapid pace of nervous system changes (Holmes and Ben-Ari, 2001), but at the same time, it makes the brain highly vulnerable to seizures and status epilepticus (Volpe, 2008). Furthermore, the basis of these seizures (e.g., seizures as a consequence of hypoxic–ischemic encephalopathy—Pressler et al., 2021) appears to be different from that of older children and adults, rendering standard treatments (phenobarbital is recommended by the Expert Group—Pressler et al., 2023) mostly ineffective (Silverstein and Jensen, 2007). In the present study, we have attempted to elucidate the neurochemical mechanisms underlying enhanced excitability. Our hope is to better understand neonatal development, which we can then use to design better treatments for neonatal seizures.

We utilized the experimental model of postictal potentiation (PIP) to study these underlying mechanisms. This model was developed in our laboratory (Mareš et al., 2002; Mareš and Kubová, 2015), utilizing 12-day-old baby rats whose state of neocortical development closely mimics that of human full-term newborns (Romijn et al., 1991). It is based on the observation that an electrical seizure (afterdischarge or AD) elicited by stimulation of the cortex in these baby rats is followed by a period of enhanced neuronal excitability during which a second stimulus will elicit a longer electrical seizure (AD). This phenomenon is unique to this period of development—in older animals, an AD is followed by several minutes of postictal depression, during which a second stimulus elicits no AD or a shorter AD. We believe that exploring the neurochemical mechanisms underlying PIP is likely to provide insights into the mechanisms underlying the unique neuronal hyperexcitability of the immature brain.

In the present study, we explored which neuronal receptors mediate PIP. Our results strongly suggest a primary role for NMDA receptors, particularly those containing the GluN2B subunit. PIP was abolished or diminished by the non-specific NMDA receptor antagonists, dizocilpine and memantine. More significantly, when specific NMDA receptor antagonists were tested, PIP was abolished dramatically by the GluN2B antagonist, ifenprodil, but was unaffected by the GluN2A antagonist, PEAQX.

This result makes sense given the developmental progression of NMDA receptors in the nervous system. NMDA receptors are tetramers composed of different subunits (Paoletti et al., 2013). There are always two GluN1 subunits, with an additional two subunits chosen from six possibilities: four different GluN2 subtypes and two different GluN3 subtypes. Most often, there are two GluN2A or GluN2B subunits.

The predominant GluN2 subtype, however, varies during development (Wenzel et al., 1997; Liu et al., 2004). In immature animals, there is a predominance of GluN2B subunits, but subsequently, there is a progressive increase in the abundance of GluN2A subunits, which predominate in adult animals.

The characteristics of receptors with GluN2B subunits differ from those of receptors with GluN2A subunits (Paoletti et al., 2013). Most significantly, the GluN2B subunits confer longer channel opening times, stronger calcium influx current, and slower deactivation. These characteristics very likely contribute to the enhanced neuronal excitability of the immature brain and strongly suggest that the presence of the GluN2B subunit is a major factor underlying PIP in immature animals.

In our experiments, when we inhibited receptors with the GluN2B subunit with ifenprodil, only receptors with the GluN2A subunit were active, mimicking the situation in adult animals. As a consequence, PIP disappeared and was replaced by postictal depression.

The role of AMPA receptors in PIP appears to be more complicated, but our data are in agreement with their role in status epilepticus (Joshi and Kapur, 2018; Leo et al., 2018). As mentioned before, following the initial pair of ADs in our experimental protocol, there is long-lasting potentiation. Ten minutes after T1, the length of the C2 AD has not returned to baseline, and it is occasionally longer than the T1 AD. Our data suggest that AMPA receptors may play a role in this prolonged potentiation.

This was most evident in the action of IEM 1460. The prolonged potentiation following T1 was selectively abolished by this drug. C2 was significantly shorter than T1 and, in fact, was no different from C1, indicating a return to baseline excitability. On the other hand, T2 continued to show immediate PIP 1 min after C2.

NBQX showed a similar effect at the low dose of 7.5 mg/kg—C2 was significantly shorter than T1, and T2 was not different from C2. Therefore, the prolonged potentiation was abolished, but the immediate potentiation was preserved. The results from higher doses of NBQX, however, complicate this interpretation. At 15 and 30 mg/kg, the effect on ADs was profound—both C2 and T2 were nearly abolished. All forms of potentiation were essentially eliminated, and overall neuronal excitability was depressed.

IEM 1460 is a selective antagonist of AMPA receptors lacking a GluA2 subunit, suggesting that this type of receptor may play a significant role in the prolonged postictal potentiation exhibited by immature animals. Like NMDA receptors, AMPA receptors are heteromeric tetramers composed of combinations of four subunits (GluA1-GluA4) (Gregor et al., 2017). Furthermore, like NMDA receptors, the subunit composition varies during development. In particular, immature animals have a greater abundance of AMPA receptors lacking the GluA2 subunit (Kumar et al., 2002). This absence of a GluA2 subunit markedly alters the functional characteristics of the AMPA receptor. Most significantly, it becomes Ca2 + permeable (Geiger et al., 1995), which may contribute to increased neuronal excitability in the immature brain.

## Conclusion

The present study indicates that postictal potentiation in the immature brain is due to its unique glutamate receptor composition. In particular, we found that PIP is dependent on the presence of NMDA receptors containing the GluN2B subunit. This subunit is uniquely predominant in the neonatal period, making it a likely candidate for mediating the enhanced excitability evident at this stage of development. As such, it should be considered an attractive target for the design of drugs to treat neonatal seizures.

Our data also indicate that AMPA receptors lacking the GluA2 subunit may contribute to neonatal neuronal hyperexcitability and increased seizure susceptibility. Specifically, these receptors appear to be involved in prolonging hyperexcitability following an AD. Such prolonged excitation may underlie the increased susceptibility of neonates to recurrent seizures and status epilepticus. Again, because the absence of the GluA2 subunit is a predominant characteristic of neonatal AMPA receptors, antagonists targeting this receptor subtype might offer a means of treating neonatal status epilepticus.

In addition, prolonged postictal potentiation may play a role in other phenomena associated with neonatal seizures. It is well known that neonatal seizures lead to a greater incidence of epilepsy and cognitive deficits later in life (Volpe, 2008; Silverstein and Jensen, 2007). Jensen et al. (1992) have developed a model of hypoxic seizures in 10-day-old rats that mimics many of the long-term postictal sequelae (Mikati et al., 2005). Interestingly, they have implicated AMPA receptors in these changes (Rakhade et al., 2008). Therefore, developing drugs that target AMPA receptors lacking the GluA2 subunit may not only be useful for treating acute neonatal seizures, especially recurrent seizures, but also for attenuating some of the detrimental long-term consequences of neonatal seizures.

The high therapeutic potential of NMDA and AMPA receptor antagonists is blocked by serious side effects, including effects on memory and cognition. The only NMDA receptor antagonist used in patients is low-affinity antagonist memantine in the treatment of Alzheimer‘s disease. This antagonist does not exhibit serious side effects. Our results demonstrate the importance of NMDA as well as AMPA receptors in the high seizure susceptibility in the immature brain and in necessity to find new antagonists of these receptors without severe side effects.

## Data Availability

The original contributions presented in the study are included in the article/supplementary material, further inquiries can be directed to the corresponding author.

## Supplementary material

Supplementary Figure 1 Schematic presentation of the experiments. C1 and C2 are conditioning stimulation and afterdischarges, T1 and T2 testing ones. In the graph are presented intervals between stimulations.

Supplementary Figure 2 Effects of MTEP on postical potentiation. Data are presented as box plots (the sample median and the 25th and 75th percentile) with whiskers (min and max). Data for presented ADs are in differeny colours. Individual values are presented as circles. x-axis – the four ADs (intervals between C and T are 1 min, between T1 and C2 10 min); y-axis – duration of ADs in seconds. Asterisks denote statistical significant difference between corresponding C and T values. Data are presented as box plots (the sample median and the 25th and 75th percentile) with whiskers (min and max). Data for presented ADs are in differeny colours. Individual values are presented as circles. Lower part of figure—table with statistical data. From left to right: drug; number of animals; dose; result of One Way ANOVA; *p*-value; *post-hoc* comparisons of all four pairs of ADs (value *q*–significant values in red).
