# Supplementary figures and images for: NMDA receptors play an important role in postictal potentiation in immature rats
Source: Front Synaptic Neurosci. 2026 Jun 9;18:1742164. doi: 10.3389/fnsyn.2026.1742164 (PMC13287056; doi:10.3389/fnsyn.2026.1742164)

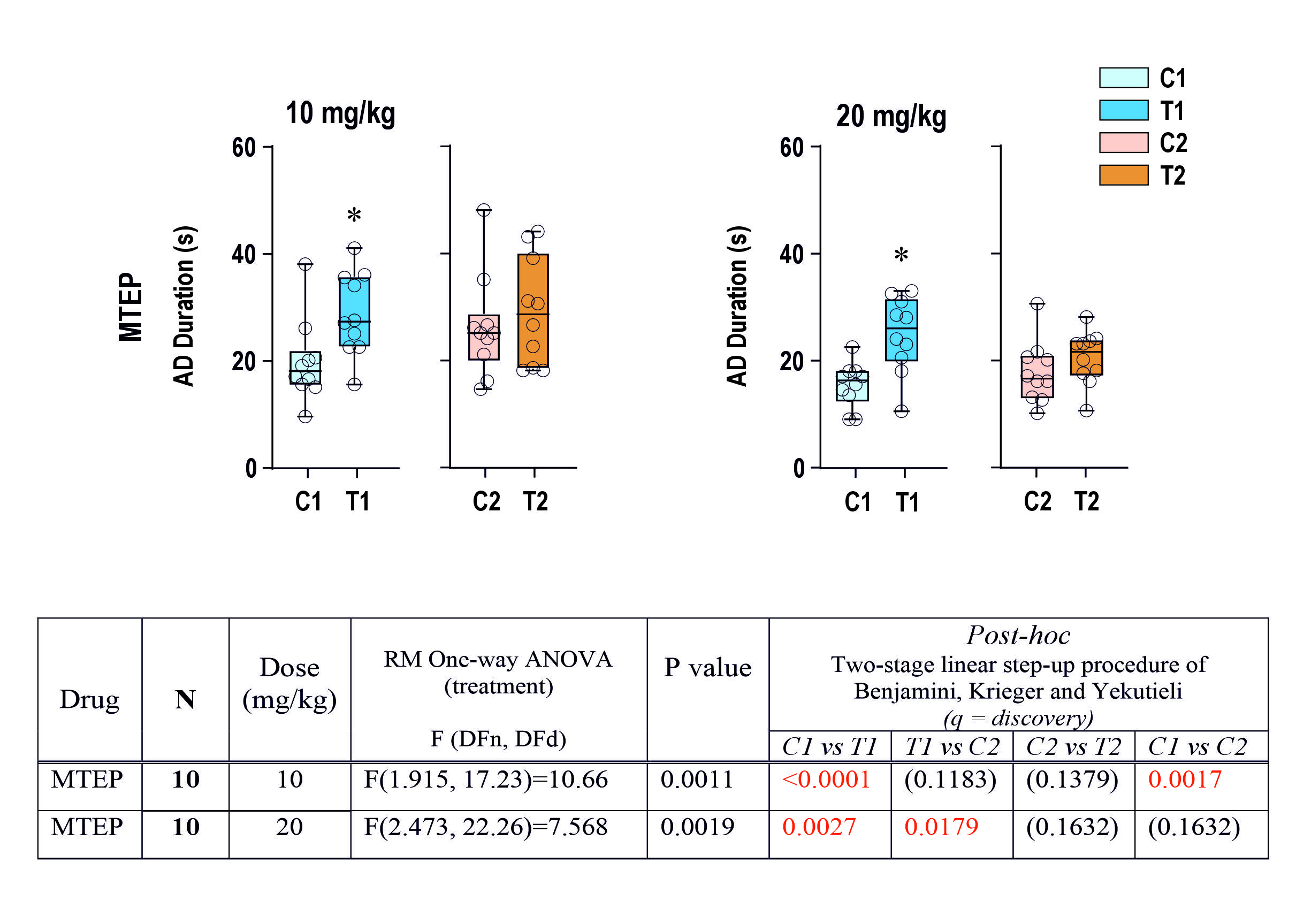

Supplement: Supplementary Figure 1 — Schematic presentation of the experiments. C1 and C2 are conditioning stimulation and afterdischarges, T1 and T2 testing ones. In the graph are presented intervals between stimulations. [file Image_1.JPEG]

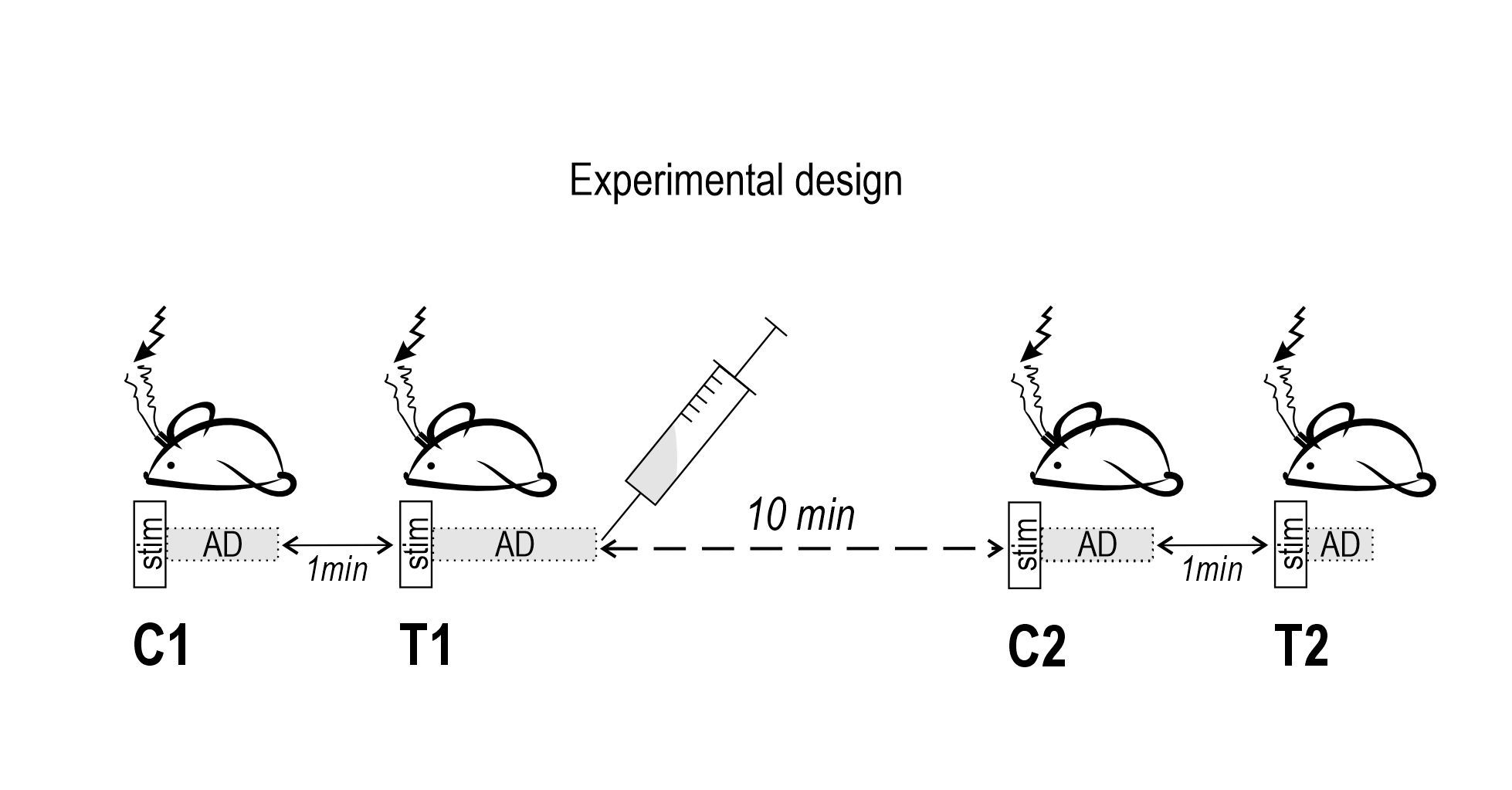

Supplement: Supplementary Figure 2 — Effects of MTEP on postical potentiation. Data are presented as box plots (the sample median and the 25th and 75th percentile) with whiskers (min and max). Data for presented ADs are in differeny colours. Individual values are presented as circles. x-axis – the four ADs (intervals between C and T are 1 min, between T1 and C2 10 min); y-axis – duration of ADs in seconds. Asterisks denote statistical significant difference between corresponding C and T values. Data are presented as box plots (the sample median and the 25th and 75th percentile) with whiskers (min and max). Data for presented ADs are in differeny colours. Individual values are presented as circles. Lower part of figure—table with statistical data. From left to right: drug; number of animals; dose; result of One Way ANOVA; p-value; post-hoc comparisons of all four pairs of ADs (value q–significant values in red). [file Image_2.JPEG]
